# Multimodal Assessment of Adolescent Coping with Family Conflict Incorporating Video-Mediated Recall Methodology

**DOI:** 10.1007/s10802-025-01290-5

**Published:** 2025-01-31

**Authors:** Kelly H. Watson, Rachel E. Siciliano, Allegra S. Anderson, Abagail E. Ciriegio, Lauren M. Henry, Meredith Gruhn, Allison Vreeland, Sofia Torres, Tarah Kuhn, Jon Ebert, Bruce E. Compas

**Affiliations:** 1 Department of Psychology and Human Development, Vanderbilt University, Nashville, TN USA; 2 Vanderbilt University Medical Center, 1161 21st Avenue S, A-0118 MCN, Nashville, TN USA

**Keywords:** Coping, Video-mediated recall, Adolescence, Internalizing, Externalizing, Conflict

## Abstract

The strategies adolescents use to cope with stress are key determinants of psychological adjustment. Research has most often utilized questionnaire methods to assess coping, which can be limited by recall bias and broad time frames. This study used a novel application of video-mediated recall methodology to assess adolescent coping during discussion of a family conflict. We examined associations between coping, observed emotions and behavior, and internalizing and externalizing symptoms. Caregiver-adolescent dyads (*N* = 89; ages 10 to 15) completed questionnaires on adolescent coping, family conflict, and symptoms of psychopathology. Caregiver-adolescent dyads were videorecorded during a 10-min conflict task. Adolescents then participated in a video-mediated recall procedure to self-report their use of coping strategies while reviewing segments of the conflict task. In addition, video recordings were coded for adolescent emotions and behaviors. Bivariate correlations revealed modest correspondence between questionnaire and recalled reports of in-the-moment adolescent coping strategies. In-the-moment coping was associated with observed and reported emotional and behavioral problems across strategies. In multivariate analyses, questionnaire reports of coping were significantly associated with questionnaire reports of internalizing and externalizing psychopathology, while in-the-moment coping responses were uniquely associated with observed emotions and behaviors. Differences in questionnaire and laboratory measures of coping underscore the need for comprehensive assessment to capture the complexity of coping in adolescence and their unique influence on emotions and behaviors and suggest that questionnaire measures may be sufficient to understand associations with global reports of symptoms. The conceptual, methodological, and clinical implications of the present study are discussed.

A central feature of risk and resilience in adolescence involves individual differences in the strategies used to cope with stressful events. Coping can be conceptualized as the thoughts and behaviors that aim to regulate internal and external experiences in the context of a stressor (Algorani & Gupta, 2021; Compas et al., 2001). The developmental trajectory of coping evolves significantly from early childhood through adolescence (Skinner & Zimmer-Gembeck, 2007). While young children largely rely on behavioral strategies (e.g., distraction, avoidance) and external support from their caregivers to help them manage distress, early adolescence is marked by rapid emotional and cognitive developmental changes that increasingly allow for the use of complex and autonomous coping strategies. Adolescence is a critical developmental period for understanding which coping strategies are effective in reducing risk for psychopathology, as the incidence of many symptoms and disorders rise considerably during this time (Dalsgaard et al., 2020). Over four decades of research has identified specific coping strategies in adolescence (e.g., avoidance, denial) that are related to both internalizing and externalizing symptoms (Compas et al., 2017; Zimmer-Gembeck & Skinner, 2011). However, a significant challenge facing coping researchers involves the identification of optimal methods to measure the ways adolescents cope with stress, particularly in ecologically relevant contexts. The aim of the current study is to examine the use of a novel laboratory methodology to measure how adolescents cope with a common source of stress: family conflict.

The current study is guided by a control-based model of coping in which strategies are structured along three categorical coping factors: (1) primary control, (2) secondary control, and (3) disengagement (e.g., Connor-Smith et al., 2000; Weisz et al., 1994). This coping model has been confirmed and validated in previous studies across a wide range of clinical and non-clinical adolescent samples (e.g., Wadsworth et al., 2004; Xiao et al., 2010). Primary control coping involves efforts to change the stressful situation or one’s response to it through the strategies of problem-solving, emotional modulation, or emotional expression. Secondary control coping involves efforts to adapt to the stressful situation or one’s response to it through the strategies of acceptance, cognitive reappraisal, positive thinking, or distraction. Extensive research has shown that primary and secondary control coping strategies are generally associated with lower levels of internalizing and externalizing symptoms (Compas et al., 2017). Disengagement coping involves efforts to evade the stressful situation or one’s response to it through the strategies of avoidance, denial, or wishful thinking. Broadly, research has shown that disengagement strategies are associated with higher levels of internalizing and externalizing symptoms (Compas et al., 2017; Richardson et al., 2021; Seiffge-Krenke & Klessinger, 2000; Watson et al., 2023).

The predominant methodology for measuring coping responses in adolescence is the use of questionnaires in which adolescents and/or caregivers are asked to reflect on what the adolescent typically does in response to stress (Compas et al., 2014, 2017). Over 80 coping questionnaires have been developed and reported in the literature, offering advantages such as being relatively quick, accessible, and cost effective. However, questionnaires are also limited by concerns regarding recall accuracy, social desirability, and shared method variance in assessments of coping and psychological symptoms (Compas et al., 2017). Additionally, young adolescents may have limited introspective capabilities, which may make it challenging for them to respond to questions about their typical coping behaviors, particularly on structured questionnaires that often require advanced verbal skills and abstract thinking. To address this developmental limitation, researchers often adopt a multi-informant assessment approach to integrate insights from caregivers and adolescents to more comprehensively evaluate children’s coping efforts (De Los Reyes et al., 2022; Hunsley & Mash, 2007). Lastly, while questionnaires can provide valuable information on the relationship between coping behaviors and psychological symptoms over an extended time period (e.g., the previous 6 months), they fail to capture the effectiveness of specific coping strategies in regulating emotions and behaviors in a shorter time window (e.g., over several minutes; Duvenage et al., 2019).

Given the limitations of questionnaire methods, alternative approaches to the measurement of coping in childhood and adolescence have been used, such as direct observation in the laboratory (Silk et al., 2006), ecological momentary assessment (EMA; Smith et al., 2023), neuroimaging tasks (McRae et al., 2012a, 2012b), and experimental paradigms (Bettis et al., 2019). While direct observations have the potential to provide more ecologically valid contexts than questionnaires that instruct participants to reflect broadly on previous stressful experiences, these studies have often relied on artificial stressors (e.g., stock photos), hypothetical scenarios, or outside observers to code and infer participants’ regulatory strategies, which limits access to covert cognitive strategies (e.g., Kliewer et al., 2006; Silk et al., 2006). To increase ecological validity, a growing body of research has used EMA methods to capture coping in real-time as stressors naturally occur. However, EMA can be burdensome for participants and lead to missing data, large volumes of complex data are produced that need to be synthesized and analyzed, and participants may not experience enough stressors during the study period to comprehensively assess their coping responses (Shiffman et al., 2008). Lastly, neuroimaging tasks and experimental paradigms try to elicit participant distress from standardized videos or images and instruct the participant to use a specific regulation strategy in response (e.g., cognitive reappraisal) to assess the effectiveness of the strategy on reducing distress. While these tasks may help understand the effectiveness of a particular coping strategy, they do not provide insight into how the participant responds in a real-life situation and how that subsequently relates to their emotional and behavioral functioning (Seeley et al., 2015). The use of controlled laboratory paradigms with personally relevant stressors may provide more stringent evidence for the role of coping as a mediator and moderator of stress and psychopathology (Troy et al., 2023).

The application of video-mediated recall (VMR) procedures to the study of coping strategies offers an innovative paradigm to assess how adolescents respond in the context of a real stressor as it unfolds as well as an understanding of how their responses relate to both observed and reported emotions and behaviors. VMR is a laboratory procedure in which participants view segments of a video recording of their interpersonal interaction and provide accounts of their emotions and cognitions (e.g., self-talk, attributions) during the interaction, which are later categorized by a trained coding team (Welsh & Dickson, 2005). VMR methods have been previously used in research with individuals, couples, caregiver-child dyads, and families to assess emotions and cognitions (e.g., Galliher et al., 2004; Gottman & Levenson, 1985; Henry et al., 2023; Lodge et al., 2000; Lorber, 2007; Schulz & Waldinger, 2004; Siciliano et al., 2023). Research has shown that participant VMR ratings are significantly associated with a variety of outcomes, including symptoms of psychopathology (e.g., Gunlicks-Stoessel & Powers, 2008), parenting behaviors (e.g., Lorber & O’Leary, 2005), psychophysiology (e.g., Lorber, 2007), relationship quality (e.g., Galliher et al., 2004), and parenting stress (e.g., Ohr et al., 2010). To date, researchers have not applied VMR to assess coping processes in adolescence.

VMR provides a myriad of promising innovative features to study coping in adolescence that minimizes concerns commonly associated with traditional coping assessments, including shared method variance, poor insight, low ecological validity, and retrospective recall. Developmentally, VMR may be well-suited for the abilities of adolescents, as it allows them to openly describe their thoughts and behaviors during a personally relevant stressor without constraining their responses to fit into a particular theoretical model of coping; experts in coping can subsequently code and categorize the responses. This method improves ecological validity and provides insight into participants’ covert experiences that are unable to be assessed through direct observation. Moreover, allowing the participant to view a videorecording of the interaction serves as a visual cue that may be especially beneficial for adolescents. This approach decreases the reliance on memory by replaying the stressor for the adolescent that occurred moments earlier. Additionally, VMR has the potential to advance our understanding of the temporal dynamics of coping effectiveness. Unlike traditional questionnaire methods that require participants to retrospectively recall and abstractly reflect on their use of pre-defined coping strategies over extended periods and across varied situations, VMR assesses participants’ current use of coping strategies in response to a specific situation. This method allows for the examination of the relationship between coping strategies and the intensity of emotions and behaviors in a short time frame (Duvenage et al., 2019). Lastly, VMR provides a potential clinical tool to facilitate teaching coping strategies to adolescents by offering the adolescent concrete examples and personalized feedback based on their own experiences and behavior. It is promising that several interventions have employed video-coaching procedures to improve parenting behaviors and attitudes (Fukkink, 2008), yet this methodology has not yet been applied in interventions to target personal coping processes.

The aim of the current multi-method, multi-informant study was to examine a novel application of VMR to assess adolescents’ use of primary control, secondary control, and disengagement coping strategies during a real-time family conflict and the associations of coping with emotional and behavioral problems. Adolescent coping was assessed with caregiver and adolescent questionnaire reports and the novel VMR methodology. Adolescent emotional and behavioral functioning were assessed with caregiver and adolescent questionnaire reports and direct observations (Melby & Conger, 2001). We focused on early adolescence given the significant developmental changes that begin to occur during this period. This stage is characterized by the emergence of advanced cognitive abilities that facilitate the use of more complex and autonomous coping strategies along with a heightened risk for psychopathology. Further, as adolescents strive for greater autonomy from their parents, they may experience greater conflict within the family. First, we hypothesized that adolescent VMR coping responses would be associated with questionnaire reports of adolescents’ coping responses. Second, we hypothesized that VMR primary control coping and secondary control coping responses would be significantly associated with lower observed and reported emotional and behavioral problems while VMR disengagement coping would be significantly associated with greater observed and reported emotional and behavioral problems. Lastly, we hypothesized that VMR coping would be a stronger predictor of observed emotions and behaviors in the family conflict task than questionnaire reports of coping. To our knowledge, this is the first study to use an observational VMR methodology to assess adolescent coping responses.

## Materials and Methods

### Participants

The sample included 97 adolescents (46% female) between the ages of 10 and 15 (*M* = 12.22, *SD* = 1.68) and their caregiver (89.7% female; *M* age = 42.04 years). Families that did not have complete data were excluded from the present analyses (*N* = 8). Data were missing on the questionnaires of coping (*n* = 2), the observational task (*n* = 2; one family did not complete the task and there was a technical error on the recording for another family), and VMR (*n* = 4). There were no differences between those with missing data and those with complete data on caregiver or adolescent age, adolescent internalizing or externalizing symptoms, or caregiver depression or anxiety symptoms. The final analytic sample included 89 adolescents (52% female) 10 to 15 years old (*M* = 12.20, *SD* = 1.71) and their caregiver (89% female; *M* age = 41.27 years). Adolescents identified as 74% White, 14% Black or African American, 6% Asian, and 6% identified as having a mixed racial/ethnic background or “other”. Caregivers identified as 78% White, 14% Black or African American, 5% Asian, 3% identified as having a mixed racial/ethnic background or “other”. Caregivers’ level of education varied, with a majority (74%) reporting at least a college degree. Most caregivers were married or living with a partner (73%). The median annual household income of the sample was $72,000.

### Procedure

Participants were invited to participate in a study designed to better understand the role of stress and emotions in the lives of families. The sample was recruited through a variety of sources, including university listservs, outpatient services, word of mouth, and adoption services. Participants who expressed an interest in the study were contacted and screened over the phone by trained research assistants to determine eligibility. Exclusion criteria included a diagnosis of autism spectrum disorder in the adolescent or a diagnosis of schizophrenia in the caregiver or adolescent due to the differences in emotional and social functioning associated with these conditions (Downs & Smith, 2004; Hooker & Park, 2002). The caregiver-adolescent dyad had to live with each other for at least 50% of the time in the previous six months, and all participants had to be fluent in English. Eligible adolescents and caregivers were invited to participate in an in-person visit that included questionnaires, a 10-min caregiver-adolescent conflict task, and a video-mediated recall procedure.

Participants completed a battery of questionnaires on REDCap, a secure web-based platform (Harris et al., 2009). Dyads were placed in a private room with a video camera and instructed to discuss the source of conflict, describe how they each felt, and try to resolve the source of conflict. Immediately following the conflict task, adolescents re-watched the middle four minutes of the conflict task while in a private room with an audio recorder. The video was automatically paused every 30 s and instructions on the screen prompted participants to answer: “What was going through your mind?” The adolescents had 20 s to respond into the audio recorder (e.g., Halford & Sanders, 1988). After the time elapsed, the next 30-s video clip started automatically. The same procedure was repeated for a total of eight 30-s video clips. All study procedures were approved by the Vanderbilt University Institutional Review Board (#181531). Caregivers provided written informed consent and adolescents provided written informed assent prior to participation and were compensated for their time ($100 for the caregivers and $50 for the adolescents).

### Measures

#### Adolescent Coping

Caregivers and adolescents completed the 57-item Responses to Stress Questionnaire – Family Stress version (RSQ; Connor-Smith et al., 2000) to assess how adolescents cope with family stress. The RSQ has a well-established factor structure of coping (i.e., primary control, secondary control, disengagement) confirmed in factor analytic studies and has been replicated with diverse samples and stressors (e.g., Wadsworth et al., 2004): primary control coping (PCC; i.e., emotional modulation, emotional expression, problem-solving), secondary control coping (SCC; i.e., acceptance, cognitive reappraisal, distraction, positive thinking), and disengagement coping (DC; i.e., avoidance, denial, wishful thinking). Proportion scores were calculated by dividing the total score for each coping factor by the total score obtained on the RSQ, which is the standard approach used for scoring the measure (Compas et al., 2006; Connor-Smith et al., 2000; Wadsworth et al., 2004) to control for response bias in item endorsement (Vitaliano et al., 1987). Composites of adolescents’ coping were created for each coping factor by creating standardized scores of the caregiver- and self-report and computing the mean. The RSQ has shown good convergence of adolescent and caregiver reports (e.g., Compas et al., 2006). In the current sample, adolescent and caregiver coping reports were significantly correlated on all three factors (*r* = 0.50 for PCC, *r* = 0.39 for SCC, and *r* = 0.34 for DC; all *p’s* < 0.001). Internal consistencies of adolescent and caregiver reports of coping were acceptable: α = 0.74 and 0.77 for PCC, α = 0.79 and 0.83 for SCC, and α = 0.79 and 0.75 for DC, respectively.

#### Video-Mediated Recall

A coding system to assess use of primary control, secondary control, and disengagement coping strategies in the observational paradigm was developed as part of the current study. The aim of the coding system was to capture the strategies adolescents used to cope *in the moment* and is consistent with the conceptual model of coping reflected in the RSQ (see Table 1 for definitions and examples). Trained research assistants transcribed the eight verbal responses from each participant that were audio recorded during the study visit into written transcripts. A team of raters then categorized the responses for each of the eight intervals and indicated the presence of each coping factor (yes/no): primary control coping, secondary control coping, and disengagement coping. If the participant’s response during the 20-s interval did not indicate a coping strategy, then they were given a “no code” for that section. All transcripts were coded by two independent raters. Raters met to compare their coping codes and reach a consensus on any discrepant codes. Reliability of the coders was excellent (92% overall agreement; PCC: 88% agreement; SCC: 92% agreement; DC: 94% agreement). Guided by the RSQ conventional scoring (Connor-Smith et al., 2000), a proportion score was calculated for each coping factor by dividing the total score for that coping factor by the total coping score.

**Table 1:** Video-mediated recall coping categories: definitions & examples

Coping Category	Definition	Examples
Primary Control	Active efforts to change the source of stress or one’s emotional response. With regard to the source of stress, it includes active efforts to change, improve, or resolve the stressful situation. With regard to one’s emotional response, it involves direct attempts to express, modify, or alter one’s own emotion(s). This includes efforts to understand one’s emotion, control the expression of the emotion, or alter the intensity of the emotion.	1. Trying to reach a win–win situation with him. 2. I know it is important to her so I am working towards a compromise. 3. I am identifying all of the resources that we have that are not so expensive. 4. I was listening to her side of the story and trying to just keep my cool. 5. Here I am trying to think of ways to feel less angry in the moment. 6. I was trying to understand what she was talking about at this point.
Secondary Control	Efforts to adapt to the source of stress or one’s emotional response. Specifically, it includes intentionally acknowledging, experiencing, or affirming the situation or one’s emotional response without attempting to change it. It also includes focusing on the good in the situation or reinterpreting the meaning of a stressor/emotion. It also refers to efforts to temporarily direct attention to something different from the stressor or one’s emotional reaction to it.	1. Maybe this is just how it is going to be. 2. I know that he thinks I am being unfair, and I told myself we may just not see eye-to-eye on it. 3. I know this is something we will have to deal with. 4. I am realizing that I may not have control over their divorce. 5. Once she said that I thought maybe I have been blowing this out of proportion. 6. I was stressed in the moment so I was thinking about soccer practice later. 7. I was thinking about how my mom actually does leave a lot of mess around the house, which I understand because she is pretty anxious so it is not a big deal.
Disengagement	Efforts to evade or bypass a stressor either physically, emotionally, or cognitively. In addition, it includes conscious refusal to accept or acknowledge an experience or that something is true. It may also refer to a desire for the situation to resolve itself or for someone else to fix it without taking steps toward changing it or accepting it.	1. I was just looking at the wall 2. I did not want to talk about it. 3. I was wondering when the task would be over. I can’t wait for this to be over. 4. I was not listening to what she was saying. 5. I was not thinking anything right here. 6. If I just sit back and do not say anything this whole issue will resolve itself. 7. I was trying not to feel anything here.

#### Internalizing and Externalizing Symptoms

Caregivers completed the Child Behavior Checklist (CBCL) and adolescents completed the Youth Self-Report (YSR) to assess adolescents’ internalizing and externalizing symptoms over the previous six months (Achenbach & Rescorla, 2001). The CBCL is a 118-item caregiver-report and the YSR is a 112-item self-report of children’s emotions and behaviors based on rating the accuracy of statements on a 3-point Likert scale (0 = *not at all true* to 2 = *very true*). Composite scores of caregiver and adolescent reports for the Internalizing Problems and Externalizing Problems scales were created by computing a standardized score for each scale of the caregiver and adolescent report and then computing the mean. Internal consistency reliabilities for caregiver- and adolescent-report were α = 0.92 and α = 0.88 for the Internalizing Problems scale and α = 0.94 and α = 0.88 for Externalizing Problems scale, respectively.

#### Observed Emotions and Behaviors

Adolescent observed negative affect and behavioral problems were coded using the Iowa Family Interaction Rating Scale (IFIRS), a macro-level coding system designed to code observed interactions at both the individual and dyadic level. Each code is rated on a 9-point Likert scale from 1 (*not at all characteristic*) to 9 (*mainly characteristic*) based on the frequency, intensity, and duration of verbal and nonverbal behaviors, affect, and tone of voice. The IFIRS system has been validated through correlational and confirmatory factor analysis (Alderfer et al., 2008; Melby & Conger, 2001). Each conflict task was double-coded by trained graduate and undergraduate research assistants who independently watched the task five times before rating each code on the 9-point Likert scale. When both raters completed coding the task, they met to compare their codes and reach a consensus on any discrepant codes (i.e., codes that differed by two or more points from each other). If the coders differed by one point, the higher code was given. This is the conventional approach used in the coding system to determine consensus codes (Melby & Conger, 2001). Reliability of the coders was acceptable (85% agreement). Following procedures used previously with IFIRS (e.g., Watson et al., 2023), a composite score for observed negative affect and observed behavioral problems were created. The observed negative affect score was the sum of the scores for Sadness and Anxiety. The observed behavioral problems score was the sum of the scores for Defiance, Antisocial, and the reversed score of Warmth.

#### Family Conflict

Caregivers and adolescents completed an adapted version of the Issues Checklist (Robin & Foster, 1989) to identify a source of conflict in the previous four weeks. The Issues Checklist lists 44 different issues that can be a source of conflict in a caregiver-adolescent relationship (e.g., doing homework; screentime; lying; talking back to caregivers). For each item, the participant indicates whether they talked with their caregiver/adolescent during the previous 4 weeks about it, and if so, the participant rates how they felt discussing the topic from 1 (*calm*) to 5 (*angry*). The issue rated as most upsetting by both the caregiver and adolescent was chosen as the topic of discussion for the conflict task. If the caregiver or adolescent did not rate the same topic highly, the research assistant selected the topic that was rated highest by the adolescent. A total score was also calculated by adding up the total score for the Issues Checklist with a score that ranged from 0 to 220.

### Data Analytic Approach

All analyses were conducted using SPSS (29th edition). Means, standard deviations, range of scores, and skewness for all study variables were calculated (Table 2). Missing data was handled using listwise deletion, which resulted in eight adolescents being excluded from the analyses. Bivariate correlations among the variables of interest were examined (Table 3) using one-tailed criteria reflecting the directionality of hypotheses. A series of multiple linear regression analyses were conducted with internalizing or externalizing symptoms as the dependent variables to examine the main effects of coping assessed through questionnaire and VMR methods while controlling for adolescent sex and total family conflict (Tables 4 and 5). Correlational analyses were used to determine covariates in the multiple linear regression analyses.

**Table 2:** Descriptive statistics for key study variables

Variable	M	SD	Min	Max	Skewness
Adolescent Age	12.20	1.71	9.00	15.00	.19
VMR PCC^a^	.45	.35	.00	1.00	.15
VMR SCC^a^	.35	.32	.00	1.00	.54
VMR DC^a^	.14	.27	.00	1.00	2.15
RSQ PCC – Adolescent Self-Report^a^	.18	.04	.08	.35	.70
RSQ SCC – Adolescent Self-Report^a^	.25	.05	.14	.44	.51
RSQ DC – Adolescent Self-Report^a^	.16	.03	.10	.24	-.02
RSQ PCC – Parent-Report on Adolescent^a^	.19	.05	.07	.35	.24
RSQ SCC – Parent-Report on Adolescent^a^	.24	.06	.12	.37	-.13
RSQ DC – Parent-Report on Adolescent^a^	.14	.03	.08	.21	.24
IFIRS Observed Negative Affect	8.16	2.37	3.00	14.00	.05
IFIRS Observed Behavioral Problems	14.38	3.26	8.00	23.00	.30
YSR Internalizing *T* Score	54.54	10.08	32.00	81.00	.02
YSR Externalizing *T* Score	50.81	9.88	29.00	76.00	.08
CBCL Internalizing *T* Score	57.09	11.78	33.00	86.00	.21
CBCL Externalizing *T* Score	52.29	11.40	33.00	78.00	.35
Issues Checklist: Family Conflict Total Score	32.48	24.10	2.00	118.00	1.44

*N* 89, *VMR* video-mediated recall, *PCC* primary control coping, *SCC* secondary control coping, *DC* disengagement coping, *RSQ* responses to stress questionnaire, *IFIRS* iowa family interaction scale, *YSR* youth self-report, *CBCL* child behavior checklist, ^a^Values presented are proportion scores

**Table 3:** Bivariate correlation matrix among key study variables

Variables	1	2	3	4	5	6	7	8	9	10	11	12	13
1. Adolescent Age	–												
2. Adolescent Sex	.13	–											
3. VMR PCC	.12	-.05	–										
4. VMR SCC	-.02	.06	-.48^***^	–									
5. VMR DC	.04	.00	-.40^***^	-.29^**^	–								
6. RSQ PCC	-.23^*^	-.10	-.03	.17^†^	-.24^*^	–							
7. RSQ SCC	-.03	-.01	-.20^*^	.21^*^	.04	.39^***^	–						
8. RSQ DC	.24^*^	.05	.14	-.27^**^	.16^†^	-.64^***^	-.48^***^	–					
9. IFIRS Negative Affect	.05	.27^**^	-.25^**^	.25^**^	.02	-.15^†^	-.18^*^	.14^†^	–				
10. IFIRS Behavior Problems	.03	-.10	-.01	-.35**	.29**	-.18	-.01	.24*	-.20^†^	–			
11. Internalizing Problems	.08	.04	.06	-.23^*^	.01	-.51^***^	-.65^***^	.40^***^	.21^*^	.16	–		
12. Externalizing Problems	.01	.07	-.01	-.22^*^	.34^***^	-.51^***^	-.46^***^	.34^***^	.03	.24^*^	.54^***^	–	
13. Issues Checklist	-.12	.23^*^	.00	-.04	.12	-.25^**^	-.36^***^	.15^†^	.25^**^	-.01	.34^***^	.45^***^	–

*N* 89, 1 Male, 2 Female, *VMR* video-mediated recall, *PCC* primary control coping, *SCC* secondary control coping, *DC* disengagement coping, *RSQ* responses to stress questionnaire, *IFIRS* iowa family interaction scale; ***p < .001, ^**^p < .01, ^*^p < .05, ^†^*p* < .10

**Table 4:** Regression analyses predicting adolescents’ internalizing and externalizing symptoms from adolescent coping responses

DV: Internalizing Problems	*β*	*t*	*p*	DV: Externalizing Problems	*β*	*t*	*p*
Model 1	F(4,84) = 9.35^**^; adjusted ${R}^{2}$ = .28			**Model 2**	F(4,84) = 12.40^**^; adjusted ${R}^{2}$ = .34		
Adolescent Sex	-.06	-.68	.50	Adolescent Sex	-.05	-.61	.55
Issues Checklist	.24	2.51^*^	.01	Issues Checklist	.36	3.90^**^	< .001
RSQ PCC	-.45	−4.79^**^	< .001	RSQ PCC	-.42	−4.73^**^	< .001
VMR PCC	.04	.42	.68	VMR PCC	-.03	-.35	.73
Model 3	F(4,84) = 16.58^**^; adjusted ${R}^{2}$ = .42			**Model 4**	F(4,84) = 10.08^**^; adjusted ${R}^{2}$ = .29		
Adolescent Sex	.00	.05	.96	Adolescent Sex	.00	-.01	.99
Issues Checklist	.13	1.40	.17	Issues Checklist	.33	3.36^**^	.001
RSQ SCC	-.58	−6.48^**^	< .001	RSQ SCC	-.31	−3.16^**^	.002
VMR SCC	-.10	−1.22	.23	VMR SCC	-.14	−1.56	.12
Model 5	F(4,84) = 6.78^**^; adjusted ${R}^{2}$ = .21			**Model 6**	F(4,85) = 10.91^**^; adjusted ${R}^{2}$ = .31		
Adolescent Sex	-.06	-.57	.57	Adolescent Sex	-.03	-.33	.74
Issues Checklist	.30	3.07^**^	.003	Issues Checklist	.39	4.20^**^	< .001
RSQ DC	.36	3.75^**^	< .001	RSQ DC	.24	2.63^*^	.01
VMR DC	-.08	-.85	.40	VMR DC	.24	2.63^**^	.01

*DV* dependent variable, 1 Male, 2 Female, *RSQ* responses to stress questionnaire, *PCC* primary control coping, *SCC* secondary control coping, *DC* disengagement coping, *VMR* video-mediated recall; ^**^p < .01, ^*^p < .05, ^†^*p* < .10

**Table 5:** Regression analyses predicting adolescents’ observed negative affect and behavioral problems from adolescent coping responses

DV: Observed Negative Affected	*β*	*t*	*p*	DV: Observed Behavioral Problems	*β*	*t*	*p*
Model 1	F(4,84) = 4.42^**^; adjusted ${R}^{2}$ = .13			**Model 2**	F(4,84) = 1.03; adjusted ${R}^{2}$ = .00		
Adolescent Sex	.20	1.98^†^	.051	Adolescent Sex	-.11	−1.03	.31
Issues Checklist	.18	1.71^†^	.09	Issues Checklist	-.04	-.32	.75
RSQ PCC	-.10	-.93	.36	RSQ PCC	.41	−1.78^†^	.08
VMR PCC	-.24	−2.45^*^	.02	VMR PCC	-.02	-.20	.84
Model 3	F(4,84) = 5.17^**^; adjusted ${R}^{2}$ = .16			**Model 4**	F(4,84) = 3.22^*^; adjusted ${R}^{2}$ = .09		
Adolescent Sex	.21	2.10^*^	.04	Adolescent Sex	-.08	-.80	.43
Issues Checklist	.15	1.36	.18	Issues Checklist	.02	.14	.89
RSQ SCC	-.19	−1.73^†^	.09	RSQ SCC	.07	.65	.52
VMR SCC	.29	2.84^**^	.006	VMR SCC	-.36	−3.44**	< .001
Model 5	F(4,84) = 2.79^*^; adjusted ${R}^{2}$ = .08			**Model 6**	F(4,84) = 3.41^*^; adjusted ${R}^{2}$ = .10		
Adolescent Sex	.22	2.05^*^	.04	Adolescent Sex	-.10	-.95	.35
Issues Checklist	.19	1.73^†^	.09	Issues Checklist	-.06	-.53	.60
RSQ DC	.10	.95	.34	RSQ DC	.21	2.03*	.045
VMR DC	-.02	-.15	.88	VMR DC	.27	2.59^*^	.01

*DV* Dependent Variable, 1 Male, 2 Female, *RSQ* responses to stress questionnaire, *PCC* primary control coping, *SCC* secondary control coping, *DC* disengagement coping, *VMR* video-mediated recall; ^**^p < .01, ^*^p < .05, ^†^*p* < .10

## Results

### Descriptive Statistics

**Fig. 1:**
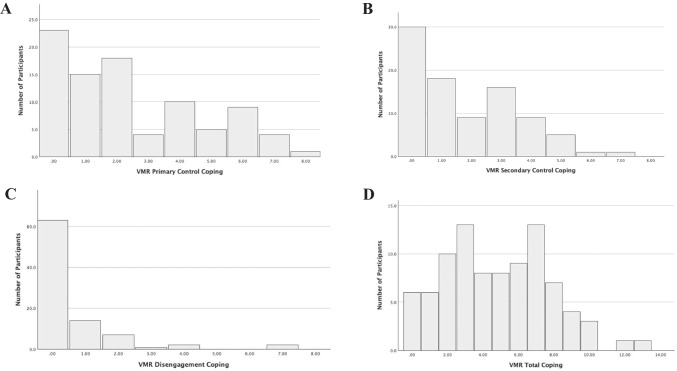
Frequency of video-mediated recall coping codes. *Note.* VMR = Video-mediated recall; The frequency of the coping factors used per participant across the eight 30-s units

Table 2 presents the descriptive statistics for all study variables. Results of the skewness analyses indicated the VMR DC code was non-normal and positively skewed (i.e., skewness > 2; Tabachnick & Fidell, 2013), which is a violation of the assumption of the normality of distribution. Therefore, it was log-transformed for all analyses to approximate normality; however, the untransformed proportion score is presented to facilitate interpretation. Most adolescents reported coping during the conflict task; only six adolescents (6.7% of the sample) did not report any coping responses during the VMR procedure. Adolescents reported most often using PCC (45% of coping responses) and SCC (35% of coping responses). While the proportion scores were used in analyses to be consistent with the scoring of the RSQ, Fig. 1 shows the frequency of the coping factors reported during the VMR procedure: PCC (Fig. 1a), SCC (Fig. 1b), DC (Fig. 1c), and total coping (Fig. 1d). Standardized composite scores from the YSR/CBCL internalizing and externalizing scales were used for all analyses; however, the *T* scores are presented in Table 2 to allow for meaningful comparisons to normative data. On the YSR and CBCL, adolescents’ externalizing symptoms approximated the normative mean. The CBCL caregiver report indicated a sample elevation on the Internalizing Problems scale (*M* = 57.09, *SD* = 11.78) as 17% of the sample had a score in the clinical range (*T* score ≥ 70); only 3% of adolescents reported internalizing symptoms in the clinical range on the YSR.

### Bivariate Correlation Analyses

Table 3 presents the bivariate Pearson correlation coefficients among the study variables.

#### VMR and RSQ Coping

There was partial support for the first hypothesis that there would be significant associations between VMR and RSQ coping responses. Specifically, there was a positive association between VMR SCC and RSQ SCC (*r* = 0.21, *p* < 0.05) and a positive trend for VMR DC and RSQ DC (*r* = 0.16, *p* < 0.10); unexpectedly VMR PCC and RSQ PCC did not show a significant correlation. There were also several significant correlations across coping strategies in the expected directions. Specifically, VMR SCC was negatively associated with RSQ DC (*r* = −0.27, *p* < 0.01) and there was a positive trend with RSQ PCC (*r* = 0.17, *p* < 0.10). VMR DC was significantly and negatively associated with RSQ PCC (*r* = −0.24, *p* < 0.05). Unexpectedly, VMR PCC was negatively associated with RSQ SCC (*r* = −0.20, *p* < 0.05). The effect sizes for the significant correlations between VMR and RSQ coping responses were small in magnitude (Cohen, 1988).

#### VMR and Observed Emotions/Behaviors

There was partial support for the second hypothesis that there would be significant associations between VMR coping responses and observed emotions and behaviors with effect sizes that were small to medium in magnitude (Cohen, 1988). In support of the hypothesis, observed negative affect was negatively associated with VMR PCC (*r* = −0.25, *p* < 0.01) and observed behavior problems were negatively associated with VMR SCC (*r* = −0.33, *p* < 0.001) and positively associated with VMR DC (*r* = 0.27, *p* < 0.05). Unexpectedly, there was a positive association between VMR SCC and observed negative affect (*r* = 0.25, *p* < 0.01) such that adolescents who were observed to exhibit greater negative affect during the conflict task with their caregiver reported greater use of secondary control coping strategies (e.g., acceptance, positive thinking).

#### VMR and Global Psychopathology Symptoms

There was partial support for the second hypothesis that there would be significant associations between VMR coping responses and questionnaire reports of internalizing and externalizing problems, with effect sizes that were small to medium in magnitude (Cohen, 1988). As hypothesized, VMR SCC was negatively associated with the YSR/CBCL Internalizing Problems (*r* = −0.23, *p* < 0.05) and Externalizing Problems (*r* = −0.22, *p* < 0.05) while VMR DC was positively associated with Externalizing Problems (*r* = 0.34, *p* < 0.001). VMR PCC was unrelated to symptoms of psychopathology.

### Multiple Linear Regression Analyses

A series of multiple linear regressions were performed to examine VMR and RSQ coping responses as predictors of adolescent global symptoms of psychopathology (Table 4) and observed emotions and behaviors (Table 5) while covarying for adolescent sex and Issues Checklist total score. Adolescent sex and the Issues Checklist total score were included as covariates because of their significant associations with the dependent variables of interest. The assumptions for multiple linear regression were tested and met for dependent variables and all models (e.g., Shapiro–Wilk tests).

#### Coping and Global Psychopathology Symptoms

Consistent with previous research, adolescent coping reported on the RSQ was significantly associated with symptoms of internalizing and externalizing problems. Specifically, primary and secondary control coping were associated with fewer symptoms while disengagement coping was associated with greater symptoms of psychopathology. VMR coping was a significant and unique predictor of symptoms in one model: VMR DC predicted greater externalizing symptoms with a small effect size (*ß* = 0.24, *t* = 2.63, *p* = 0.01) after covarying for adolescent sex and the Issues Checklist total score.

#### Coping and Observed Emotions/Behaviors

There was partial support for the third hypothesis that VMR coping codes would be stronger predictors of observed emotions and behaviors than questionnaire reports of coping, with effect sizes that were small to medium (Cohen, 1988). As hypothesized, VMR PCC uniquely predicted lower observed negative affect in the conflict task (*ß* = −0.24, *t* = −2.45, *p* = 0.02); however, it was not related to observed behavior problems. As hypothesized, VMR SCC uniquely predicted fewer observed behavior problems (*ß* = −0.36, *t* = −3.44, *p* < 0.001); however, contrary to the third hypothesis, VMR SCC uniquely predicted higher levels of negative affect during the conflict task (*ß* = 0.29, *t* = 2.84, *p* < 0.01). As hypothesized, VMR DC uniquely predicted greater observed behavior problems (*ß* = 0.27, *t* = 2.459, *p* = 0.01); however, VMR DC was not related to observed negative affect.

## Discussion

The current study examined the link between adolescent coping strategies with both proximal and distal measures of emotional and behavioral adjustment. A key feature of this study was the introduction of a novel application of VMR methodology to measure adolescent coping during a laboratory conflict task with their caregiver, combined with adolescent and caregiver-report questionnaires to provide a comprehensive assessment of adolescent coping. Overall, there was modest correspondence between VMR and questionnaire-based methods of coping assessment. Further, there was partial support for significant associations between adolescents’ coping assessed through VMR and both concurrently observed emotional and behavioral difficulties, as well as global reports of internalizing and externalizing symptoms. Below, each of the study findings are discussed in turn and implications and suggested directions for future research are detailed.

VMR has previously been used to measure in-the-moment emotions and cognitions (Galliher et al., 2004; Gunlicks-Stoessel & Powers, 2008; Lorber & O’Leary, 2005; Lorber, 2007; Ohr et al., 2010) but has not yet been applied to assess processes of coping. The VMR coding system developed in the present study was guided by a control-based conceptual coping model (Connor-Smith et al., 2000; Weisz et al., 1994) that includes primary control (PCC), secondary control (SCC), and disengagement (DC) coping efforts. The study findings indicated the observational conflict task was a sufficiently stressful context for adolescents to generate open-ended reports of all three coping types during the VMR paradigm. However, it is notable that the DC code was positively skewed, which indicates many adolescents did not report using this coping strategy during the 10-min conflict task. Given that DC involves efforts to evade a stressor or one’s emotions (i.e., strategies of avoidance, denial, and wishful thinking), the task demands may have constrained adolescents’ ability to implement DC strategies. Participants were instructed to remain in the room and discuss the conflict topic for 10 min with their caregiver, and strategies such as avoidance may not have been as feasible to use. Importantly, while participants were told they were free to end the task at any time, no families stopped the task early.

There was modest correspondence between coping strategies reported in-the-moment through VMR with those reported on the RSQ (Connor-Smith et al., 2000). Specifically, there was a significant positive association in reports of SCC across methods, which is particularly encouraging given SCC strategies involve covert cognitive processes (e.g., cognitive reappraisal, positive thinking, acceptance) that are traditionally more difficult to assess spontaneous use in a laboratory setting. There was a non-significant positive trend for the assessment of DC strategies. The absence of significant correspondence for PCC strategies across methods is noteworthy. PCC involves active efforts to alter the stressor or one’s emotional state through strategies such as problem-solving and emotional modulation. One plausible explanation for this null result is the nature of the conflict task, which instructed dyads to discuss the conflict and to try and reach a solution. The focus on finding a solution may have overrepresented how often adolescents use PCC to cope with family stress. As further evidence of this potential bias, PCC was also the most frequently endorsed coping strategy during the VMR task whereas SCC was more frequently reported on the questionnaire. Taken together, while there was modest correspondence across these two methods, the discrepancies suggest that VMR is measuring complementary yet unique insights into how adolescents cope with family conflict.

There was partial support for associations between VMR-based coping strategies with emotional and behavioral problems in bivariate analyses. As expected, PCC was associated with less observed negative affect during the conflict task. That is, adolescents who reported trying to solve the problem or regulate their emotions during the conflict task with their caregiver were observed by objective raters to display less negative affect. However, VMR PCC was not related to observed behavioral difficulties or symptoms reported on the YSR/CBCL. Notably, PCC reported on the RSQ was not significantly associated with observed emotions or behaviors, although it was negatively associated with questionnaire reports of internalizing and externalizing symptoms, which replicates previous research (Compas et al., 2017). DC was positively associated with both observed and reported behavioral difficulties, suggesting that DC in the context of family stress is a risk factor for behavioral problems in adolescence. Interestingly, there was no association of DC strategies with either observed negative affect or internalizing symptoms. While contrary to our expectation, disengagement strategies involve attempts to avoid the present experiences and inhibit emotions, and therefore may contribute to neutral affect rather than either an upregulation or downregulation of emotions (Gross, 1998).

As hypothesized, the use of SCC reported from VMR was negatively associated with observed behavioral difficulties and both internalizing and externalizing symptoms on the YSR/CBCL. These findings replicate previous research and suggest that habitual use of SCC is adaptive in the long-term, as both RSQ and VMR SCC were negatively associated with symptoms on the YSR/CBCL (Aldao et al., 2010; Compas et al., 2017). However, VMR SCC unexpectedly showed a positive association with observations of negative affect during the conflict task, suggesting that in-the-moment use of SCC is associated with greater emotional distress in the short-term. One possible explanation for this seemingly paradoxical finding is that SCC involves active efforts to adapt to the source of stressor or one’s emotions, such as through acceptance. Acceptance can be defined as a process of *allowing* emotions or experiences without trying to change them. Acceptance is a core process of change in both Acceptance and Commitment Therapy (Hayes et al., 2006) and Dialectical Behavior Therapy (Linehan, 2015) and has consistently been shown to predict better long-term mental health (e.g., Aldao et al., 2010; Ford et al., 2018). However, the active use of acceptance may not immediately reduce feelings of distress, as the individual is open to experiencing something psychologically difficult and often uncontrollable. While a small body of research has reported similar findings in experimental studies of acceptance (e.g., Boehme et al., 2019), this requires additional research. As a whole, the findings suggest that coping strategies that provide long-term psychological health benefits may not result in immediate reductions in distress, which can be important to acknowledge to individuals when teaching coping skills in a clinical context. It is important to note that SCC is a broad coping factor that also includes the strategies of positive thinking, reappraisal, and distraction. In the current study, it was not possible to disentangle the effects of specific strategies on adjustment, but this is a potential avenue for future research.

While VMR-based coping demonstrated bivariate associations with internalizing and externalizing symptoms on the YSR/CBCL, these associations were no longer significant in multivariate analyses when questionnaire reports of coping were included in the model. However, VMR-based coping strategies were uniquely associated with observed negative affect and behavioral difficulties after accounting for adolescent sex, total family conflict, and questionnaire assessments of coping. These findings suggest that in-the-moment coping strategies are more strongly linked to concurrent emotions and behaviors than questionnaire coping assessments. Further, these findings suggest that PCC strategies may be more effective in regulating negative emotions while DC is important for behavioral regulation. In contrast, greater use of SCC was linked to higher levels of concurrent negative affect (i.e., proximal distress) but lower levels of internalizing symptoms on the YSR/CBCL (i.e., distal distress), highlighting the importance of understanding the temporal dynamics of coping processes.

These findings have important implications for the measurement and understanding of coping. First, the study suggests that VMR can be a valuable tool for understanding the strategies adolescents use to cope in real-time; however, the effectiveness of coping strategies may vary depending on the task demands, stressor, emotions elicited, and the timing of assessment (Aldao, 2013; Troy et al., 2023). Further, while coping strategies are often broadly labeled as “adaptive” or “maladaptive”, the present findings suggest that effectiveness of strategies may be more nuanced, and it is critical to understand how coping processes unfold over time and in combination (Konstantinou et al., 2024). Second, research on coping has primarily used questionnaire reports to assess frequency of children’s use of a variety of coping strategies. However, it is unclear from these reports how skilled children are in using these coping strategies. While some research has examined coping ability (e.g., McRae et al., 2012a, 2012b), VMR provides a method to understand how skilled adolescents are in using coping strategies by objectively rating the effectiveness of the reported coping strategy. While beyond the scope of the present study, this is a future avenue for research. Third, future research could benefit from integrating VMR methods with other observational or physiological measures to provide a comprehensive understanding of how adolescents respond to stress. Fourth, future research should broaden the task demands to facilitate a broader use of coping strategies and enhance the utility of VMR. Lastly, it is noteworthy that the VMR methodology was uniquely associated with observed emotions and behaviors whereas questionnaire assessments of coping and symptoms exhibited stronger intercorrelations. This pattern may reflect the presence of shared method bias, suggesting future research would benefit from adopting a multi-trait, multi-method, multi-source approach to disentangle measurement biases and provide a more accurate understanding of the true relationships among variables.

The present study had several limitations. First, the study was cross-sectional and so the direction of effects cannot be determined. Second, the sample was predominantly white, middle and upper socioeconomic status, English-speaking, and enrolled mostly mothers, which may limit the generalizability of the study findings to other sociocultural contexts (Nielsen et al., 2017). It will be important for future research to include a more diverse sample of adolescents and caregivers. Third, due to missing data, 8% of the sample was excluded in the present analyses. While there were no differences in demographics or caregiver psychopathology between those with and without missing data, it is possible that the data were not missing at random. Further, our sample size was relatively small, and so caution is warranted when interpreting the precise magnitudes of associations. Larger studies are needed to confirm these results and evaluate the robustness and stability of the findings. Fourth, it is important to acknowledge that the low correspondence between methods may also represent differences in measurement periods (i.e., coping in the moment versus a wider time frame) and coverage of stressors (i.e., conflict with caregiver about one topic versus other family stressors). It will be important for future studies to assess coping using a questionnaire and VMR in reference to the same time frame and stressor to better understand the correspondence across methods. Fifth, our observed negative affect code was only a composite of sadness and anxiety. However, anger may be an important emotion to consider in the context of family conflict. Future research should assess a broader range of emotions to evaluate the utility of VMR for measuring coping. Lastly, this was a non-clinical sample, and so it will be important for future studies to examine these associations in clinical samples with greater levels of psychopathology and coping difficulties. However, these limitations were offset in part by study strengths that include the assessment of coping in real-time in the context of an ecologically valid stressor, the use of a multi-method approach to assess coping as well as emotional and behavioral difficulties, and the use of observational methods with two independent raters.

## Conclusion

The present study utilized a cross-informant, multi-method design to expand previous research on adolescent coping. A novel VMR-based paradigm was integrated with a traditional coping questionnaire method to explore the link between coping with both concurrent and global assessments of emotional and behavioral adjustment. The study findings demonstrated modest correspondence between VMR and questionnaire-based assessment of coping. While questionnaire reports of coping were more strongly associated with internalizing and externalizing symptoms reported on questionnaires, VMR methods demonstrated unique associations with concurrent emotions and behaviors. More specifically, PCC was linked to lower negative affect, DC was linked to greater behavioral difficulties, while SCC was related to *higher* concurrent negative affect but *lower* internalizing symptoms aggregated across 6-months. Taken together, these results underscore the importance of considering both timing and context when understanding the effectiveness of coping strategies. VMR methods have the potential to provide valuable insights into the temporal dynamics of coping processes and suggest that this novel paradigm is a promising method to provide complementary information to traditional questionnaire methods.

## Data Availability

The data surrounding the findings of this study are available upon reasonable request from the corresponding author.
